# EMBRACING THE QUEER ART OF FAILURE IN GERONTOLOGICAL RESEARCH AND EDUCATION

**DOI:** 10.1093/geroni/igad104.0254

**Published:** 2023-12-21

**Authors:** Sara Bybee, Austin Oswald, Vanessa Fabbre

## Abstract

Halberstam describes the queer art of failure as a performance of dissidence in which lesbian, gay, bisexual, transgender, and queer plus (LGBTQ+) people willing reject traditional conceptualizations of success. Yet, dominant theoretical frameworks in gerontology are predicated upon notions of success and productivity which may be problematic for understanding the life trajectories of LGBTQ+ people. The use of such heteronormative frameworks has implications for gerontological research in two important ways: 1) Research methods and researchers themselves may be constrained by the normative expectations placed on LGBTQ+ people and 2) Heteronormative frameworks obscure the nuance of LGBTQ+ older adults’ lived experiences and may limit important contributions to gerontological knowledge. This symposium applies the queer art of failure to examine LGBTQ+ aging scholarship that deviates from traditional research and education. Speaker one shares experiences from LGBTQ+ individuals facing dementia, using concepts that counter framing dementia as pathology and decline. Speaker two discusses how participants’ preferences for receiving research results via found poetry may reflect LGBTQ+ participants’ natural inclination to question hegemonic norms. Speaker three describes collaborating with a coalition of LGBTQ+ older adults of color on a participatory action research study, detailing how epistemic tensions shaped the research in unexpected ways. Speaker four discusses how institutional failure led to enthusiasm for LGBTQ+ curriculum development, student mentoring, and knowledge production. These presentations suggest that the inclusion of diverse conceptualizations of success and productivity should inform future aging scholarship, as they may center the experiences of historically marginalized populations such as LGBTQ+ older adults.

